# Acupuncture and Herbal Medicine for Cancer Patients 

**DOI:** 10.1155/2013/313751

**Published:** 2013-11-25

**Authors:** S. Schröder, S. Lee, T. Efferth, Y. Motoo

**Affiliations:** ^1^HanseMerkur Center for Traditional Chinese Medicine, University Medical Center Hamburg-Eppendorf, 20246 Hamburg, Germany; ^2^Department of Clinical Oncology, College of Korean Medicine, Kyung Hee University, Seoul 130-701, Republic of Korea; ^3^Department of Pharmaceutical Biology, Institute of Pharmacy and Biochemistry, Johannes Gutenberg University, StaudingerWeg 5, 55128 Mainz, Germany; ^4^Department of Medical Oncology, Kanazawa Medical University, Uchinada, Ishikawa 920-0293, Japan

## 1. Complementary and Alternative Medicine (CAM) in Cancer Care

In recent decades, cancer treatment has made remarkable progress with targeted therapy applied on targeted subpopulation [1]. Though the life expectancy of the general population is increasing, cancer is still one of the most common causes of death worldwide [2]. The sophisticated treatments are often accompanied with increased adverse events, and the survival rate is still unsatisfactory in some cancers. In advanced or recurrent cancers, the goal of cancer treatment is often not curing the disease but prolonging survival time with good quality of life. The health-related quality of life is getting value as a cancer outcome, and the effect of early palliative care is emphasized importantly [3]. Nowadays medical information is easily accessed by patients, and most of patients are looking for treatments with less side effects and all available information to prolong survival time and to improve quality of life. Therefore, complementary and alternative medicine (CAM) for cancer is increasingly being demanded by patients, and more physicians are getting interested in the use of CAM in cancer therapies.

The use of CAM in cancer therapies differs from country to country, but up to 80% of cancer patients use some kinds of CAM to support their conventional cancer treatments [4, 5]. In Southwestern China, the prevalence of Chinese herbal medicine use is up to 53.0% during cancer treatment [6], and it seems similar to that of Hong Kong [7]. In Taiwan, up to 98% of the cancer patients use any kind of CAM [8]. In Korea, 78.5% of cancer patients use CAM [9]. A Japanese study revealed that 44.6% of cancer patients use CAM [10], while 83% of cancer patients or cancer survivors use CAM treatments in Australia [11]. An European survey in 13 countries showed 35.9% of average CAM prevalence in cancer patients (range among countries 14.8% to 73.1%) [12]. In other parts of the world similar results were obtained [13]. CAM is also used by 31–84% of children with cancer [14]. This usage of CAM for cancer is more common among educated people with better health behaviour [15, 16] as well as among women [17]. In the case of female cancers, more patients use CAM; for example, 60–80% of breast cancer patients (in comparison to 50% among cancer patients in general) and 75% of female colon cancer patients use CAM [18].

Patients mostly use CAM as a complement to their conventional therapy, not as an alternative treatment [19]. Only very few patients replace conventional treatment by alternative medicine [20]. Even though the use of CAM is a fact worldwide, the view on CAM varies enormously, and the integration of CAM therapies into conventional therapies is extremely complicated in different countries.

Although the governmental support on the research on CAM for cancer therapies differs among various countries, the use of CAM for cancer patients is still debated in an ideological way by supporters and opponents of CAM. One of the causes for the reluctance of Western academia towards CAM is still the insufficient number of convincing clinical studies providing evidence for the efficacy and safety of CAM therapies. The Western concept of   “evidence-based medicine” does only poorly match to the clinical practice of CAM. Nevertheless, an increasing number of clinical studies are being conducted during the past years to gain credibility and reputation of CAM. Illustrative examples on the power of CAM have been documented in cancer research with a focus on three major fields [21]:

- acupuncture is widely applied for treating pain, a prominent side effect encountered during cancer therapy;
- reduction of severe adverse effects of standard chemotherapy;
- unwanted interactions of standard therapy with herbal medicines.

Another reason for critical opinions towards CAM regards the quality of herbal products according to international quality standards [22].

Therefore, an important question for recognition and implementation of CAM into general medical practice concerns the clinical evidence for efficacy and safety of CAM treatments.

This induces confusion and fear in patients and provides a burden for the communication between patient and doctor. Only 2.4% of patients used their healthcare professional record as primary source of information on CAM in cancer care [23] and 92% of American breast cancer patients withheld information about the CAM treatment from their medical oncologists [24]. Similar results were found in a survey in Taiwan, where more than two out of three cancer patients never informed their physicians of their CAM use [8]. Reasons for this might be the expected inflexibility of medical oncologists and patients' fear of harming the relationship with their oncologists caused by the use of CAM. It is not common for patients to have a communication between Western medicine-oriented doctors and CAM practitioners in clinical practice. However, the integrative cancer care is increasingly required by patients and recently it is getting popular in cancer treatment.

## 2. Acupuncture and Herbal Medicine for Cancer Patients

Acupuncture and herbal medicine are the most qualified CAM treatments. Their effectiveness is still under debate, although they have been used in many fields of cancer treatment and palliative care [25, 26]. The efficacy of the acupuncture and herbal treatment is not currently well recognized by the Western academia and clinical scholars, so that advantages of these therapies in treating cancer or supporting cancer treatment are not adequately reflected. One reason of this estimation is because of the quality of clinical research. Many studies still have the level of case studies [27], which seem like an anecdotal, and the generalization of CAM results might be questionable for scholars who are familiar with well-designed clinical trials.

Acupuncture and herbal medicine have been used for the treatment of cancer pain [25, 26] and for attenuation of side effects of cancer treatments. Acupuncture has been shown to be effective for chemotherapy-induced nausea and vomiting [28], as well as acupressure [29] and herbal medicine [30]. However, acupuncture has not been systematically evaluated with well-designed clinical trial for its effect on it, while new drugs got approvals from FDA for the relief of chemotherapy-induced nausea. Other studies showed an effect of acupuncture on xerostomia after radiation therapy [31, 32]. There are reports that acupuncture was effective on the chemotherapy-induced peripheral neuropathy [33] and herbal medicine was effective on the oral stomatitis [34], which lead patients to higher acceptance of Western cancer treatment with less interruption or discontinuation of therapy. Many studies were focused on the relief of cancer-related symptoms such as fatigue [35–37] and the improvement of quality of life [38].

Recently, randomized controlled trials (RCTs) have been conducted to seek evidences on acupuncture and herbal medicine [39–41]. These therapies are usually considered as supportive for major treatment, in order to attenuate side effects of surgery, radiotherapy, and chemotherapy.

One of the issues of RCTs on acupuncture and herbal medicine is how to deal with the individualized approach of treatments based on the Asian tradition. Asian herbal therapy is commonly a combination of multiple herbs; sometimes approximately up to 15 herbs are in one prescription, while a single herb already contains multiple tentative active compounds. The daily practice in acupuncture and herbal medicine with its individualized approach cannot be easily transferred into standardized controlled trials. The conclusion of systematic review studies that better qualified studies are necessary [42–44] is not so surprising. The study design to solve these issues should be developed.

Even though the effects of acupuncture and herbal medicine are still under debate and further clinical research is necessary, the clinical use of acupuncture and herbal medicine has already been recommended to control cancer-related symptoms in some of the clinical practice guidelines. According to the evidence-based guidelines of the *American College of Chest Physicians for Lung Cancer*, acupuncture has been recommended as a complementary therapy for lung cancer when pain is poorly controlled or when side effects such as neuropathy or xerostomia are clinically significant [45].

Acupuncture and herbal treatment have been used as a complementary treatment in combination with highly effective and partly aggressive Western medicine such as chemotherapy or hormonal therapy. But interactions are quite unknown, underestimated, or under debate. For instance, the herbal treatment with hormone-active herbs in patients with hormone-sensitive cells of breast or ovarian cancers is an important topic of ongoing debates [46, 47]. The interaction of acupuncture and especially herbal medicine with conventional treatments is not all known. Guidance on the safety of herbal medicine to prevent potential risks to cancer patients is necessary, but data have not yet been collected systematically in Mainland China but are now being established in Hong Kong.

In Japan, Kampo, traditional Japanese medicine, is extensively used for cancer patients as supportive measures, covered by National Health Insurance. Japanese medical doctors can prescribe both Western and Kampo drugs, knowing the natural history of diseases and the indication and limitation of Kampo. But, there are no strong recommendations on the Kampo use based on high-quality evidence in clinical practice guidelines in Japan [48]. The Japanese medical system is a unitary one, and Kampo is practiced in this system. From this point of view, the system of Japanese traditional medicine is different from those of China and Korea, where traditional medicine is generally practiced in a dual system, but in the recent years integrative approaches were developed. Japanese Kampo practitioners take advantage of this unitary system, conducting high-quality clinical practice and research. This situation consequently leads to the integrative medicine by a single doctor, whereas the integrative medicine in other countries is usually done by a Western medicine doctor and a traditional medicine practitioner. The system of Japanese Kampo medicine well fits the methodologies of modern medicine, and many clinicians utilize Kampo, accumulating evidence data. This unified situation might be an inspiring example for countries with a unitary medical system.

In Korea, the government health insurance covers only acupuncture treatment for cancer patients. In Western countries, acupuncture and herbal medicine, in spite of frequent use, for years were not in the main focus of the medical academic society. So, research in this field was limited leading in consequence to a situation that especially treatments with Asian herbs often had a lack of scientific controls. But due to increasing interest of patients and practitioners, acupuncture had partly become an integrative therapy in pain management, and, for example, in Germany the use of Western herbs as a complementary medicine is common and Asian herbs are increasingly used.

Since herbal therapy is the most commonly used CAM treatment [49], in recent years, the search for active compounds has mainly focused on Asian herbs, whereby the emphasis has been on classical product-based leads for Western drug discovery, usually performed by screening the extracts or compounds from diverse biological sources. Many *in vivo* experiments showed effectiveness on cell cultures and in animal models [50–53], but translation from bench to bedside is still a difficult challenge. This research, mainly focusing on single active compound, has been done often without regard to preexisting knowledge of the therapeutic utility of the plant source [54]. While interactions of ingredients during the preparation procedure are sometimes essential to the therapy, an extraction of the active ingredients is often not a simple task, and evidence shows that single components extracted from plants are less potent than the crude extract [55]. Scientists of many countries worldwide have tried to apply modern experiment-based research methods to isolating active compounds from herbs, characterizing their pharmacodynamic and pharmacokinetic properties and defining their molecular modes of action with limited success. This reductionist paradigm of a “single chemical entity” is not easily applicable to the multidimensional complexity of Asian herbal prescriptions. Researchers often do not use any concepts of traditional theory as the basis for their investigations on these compounds [56].

Studies on the influence of single herbs or their components on different microbiological pathways of human physiology are necessary and important, but this research is not likely to lead to single-component treatments for multifactorial diseases such as cancer. Cancer is a systemic disease of the entire body. A single-target approach has limited effectiveness, and there is evidence that a multitarget approach might be more effective [57, 58]. It seems only rational to apply a multitargeted therapy to a multifactorial disease. The realization that multicomponent medicines may have advantages over single-component drugs has a scientific foundation. The pharmacological advantages of mixtures may lie in the potentiating action of their multiple bioactive components and the advancement of individualized therapy [59, 60].

Modern research methods on a single herb aimed at isolating active compounds from herb have to be the fundament for future researches in herbal medicine. But when basic information is found and made available, experiments with herbal combinations might be the productive direction for further research to control cancer. While cancer is a multifactorial disease with diverse heterogeneous mechanisms, a combination of components might provide a promising opportunity to focus on multiple targets. Furthermore, these efforts may eventually offer an individualized approach to the treatment. Basic research on single herbs and their active compounds is still essential for the scientific understanding of traditional herbal medicine. But research should not stop at this level but continue with research on multicompounds, their interactions, and increasing or decreasing activity in combinations. Gaining knowledge from tradition might be helpful, not ending in a dead end. This approach is ambitious and time-consuming but has a chance not to fail like conventional drug discovery procedure in the field of herbal medicine in recent years.

While it is difficult to get a patent on natural products, the further interest of pharmaceutical companies might be limited. Progress in this research area can only be found in intense national as well as international cooperation, founding international joint working groups to overcome the obstacles of this sophisticated challenge.

*S. Schröder* *S. Schröder* *S. Lee* *S. Lee* *T. Efferth* *T. Efferth* *Y. Motoo* *Y. Motoo*
